# Integrative OMICS Data-Driven Procedure Using a Derivatized Meta-Analysis Approach

**DOI:** 10.3389/fgene.2022.828786

**Published:** 2022-02-04

**Authors:** Karla Cervantes-Gracia, Richard Chahwan, Holger Husi

**Affiliations:** ^1^ Institute of Experimental Immunology, University of Zurich, Zurich, Switzerland; ^2^ Institute of Cardiovascular and Medical Sciences, University of Glasgow, Glasgow, United Kingdom; ^3^ Division of Biomedical Sciences, Centre for Health Science, University of the Highlands and Islands, Inverness, United Kingdom

**Keywords:** meta-analysis, omics, bioinformatics, biomarker analysis, pathway analysis, data integration

## Abstract

The wealth of high-throughput data has opened up new opportunities to analyze and describe biological processes at higher resolution, ultimately leading to a significant acceleration of scientific output using high-throughput data from the different omics layers and the generation of databases to store and report raw datasets. The great variability among the techniques and the heterogeneous methodologies used to produce this data have placed meta-analysis methods as one of the approaches of choice to correlate the resultant large-scale datasets from different research groups. Through multi-study meta-analyses, it is possible to generate results with greater statistical power compared to individual analyses. Gene signatures, biomarkers and pathways that provide new insights of a phenotype of interest have been identified by the analysis of large-scale datasets in several fields of science. However, despite all the efforts, a standardized regulation to report large-scale data and to identify the molecular targets and signaling networks is still lacking. Integrative analyses have also been introduced as complementation and augmentation for meta-analysis methodologies to generate novel hypotheses. Currently, there is no universal method established and the different methods available follow different purposes. Herein we describe a new unifying, scalable and straightforward methodology to meta-analyze different omics outputs, but also to integrate the significant outcomes into novel pathways describing biological processes of interest. The significance of using proper molecular identifiers is highlighted as well as the potential to further correlate molecules from different regulatory levels. To show the methodology’s potential, a set of transcriptomic datasets are meta-analyzed as an example.

## 1 Introduction

Traditional data analytical approaches focus on hypothesis-driven methods to understand specific and known molecular targets. Alternatively, data-driven approaches are based on high-throughput methodologies that provide un-biased genome-wide analysis of multiple omics variables which mirrors the different layers of biological regulation of a system. Undoubtedly, knowledge generated by traditional approaches through the years is essential to contextualize and properly analyze high-throughput data (McDermott et al., 2013; Guan et al., 2020). Ultimately, data-driven approaches aim to provide a number of potential hypotheses that feed into the traditional approach cycle in order to be validated or refuted (Fernandes and Husi 2019).

Nowadays, the surge of studies based on high-throughput data analysis has led to an expansion of public repositories (i.e., GEO, ArrayExpress) that store and provide access to these data for further analyses (Clough and Barrett 2016; Athar et al., 2019). As a consequence, big data production and availability have provided novel venues/opportunities for data interpretation, data integration, statistical analysis, and therefore new hypotheses that might reveal new inferences and provide a higher molecular resolution of a determined phenotype or disease. Nevertheless, the lack of a unified system to publish the different omics data generated and to report, curate and consolidate all the different identifiers available remains a challenge (Durinck et al., 2005; McGarvey et al., 2019). Unique outcomes generated from the different high-throughput technologies and the lack of standardized approaches to analyze, integrate and interpret these heterogeneous and often incompatible data have led to the emergence of different analytic methodologies that focus on varying ways of data-interpretation.

Meta-analytic methodologies have been commonly followed in data science to collate and identify commonalities across different studies, and to rule out inconsistencies commonly found in published literature (Waldron and Riester 2016; Vennou et al., 2020). The statistical basis of these methodologies provides valuable results and gives strength to the variables that reflect an association and consistency across studies. These methodologies are based on the fact that even amongst heterogeneous studies, associations can be made. Thus, meta-analysis can lead to the identification of robust and quantifiable variables shared across studies published by different groups–despite inherent differences in such cohorts–generated through different platforms and techniques that could have been otherwise overlooked (Care et al., 2015; Cho et al., 2016; Piras et al., 2019; Winter et al., 2019). In the big-data field, the exponential growth of high-throughput data availability has highlighted the advantage to follow meta-analysis methodologies in order to increase the statistical power of the outcomes and make sense out of the great amount of data shared within the scientific community (Xia et al., 2013; Kim et al., 2017; Forero 2019; Jaiswal et al., 2020; Vennou et al., 2020).

The generation and analysis of high-throughput data are commonly focused on a single biological parameter (e.g., transcripts, proteins, or metabolites) and represent only a snapshot of what is happening in a specific molecular process. Due to the high density of available studies, several meta-analytic approaches have been developed and standardized to integrate transcriptomic data. Effect size (t-statistic combination), rank-ratio (fold-change ratio combination), Fisher’s (*p*-value combination), and vote-counting (VCS–number of reporting studies) are some of the common methods followed to perform a meta-analysis on these samples (Rikke et al., 2015; Goveia et al., 2016; Forero 2019; Shafi et al., 2019; Toro-Domínguez et al., 2020). Among the many promising applications of these approaches two stand out; namely, biomarker discovery and signaling pathway identification. The premise that biomarkers identified with computational approaches from a single high-throughput study exhibit little overlap with other studies indicates that these might represent false positives and cannot be fully trusted. Thus, meta-analyses have been long-performed with the goal to discover novel and robust biomarkers, distinguishable and consistent patterns of disease-associated deregulated genes. Statistically significant deregulated genes have been associated with several cancers and other diseases through the application of different meta-analytic approaches (Fishel et al., 2007; Xu et al., 2009; Huan et al., 2015; Cho et al., 2016; Bell et al., 2017; Piras et al., 2019; Su et al., 2019). Pathway analyses have also dominated the meta-analysis studies aiming to highlight the main deregulated processes to some extent (Kröger et al., 2016; Wang et al., 2017; Badr and Häcker 2019).

Nevertheless, due to the dynamicity of biological systems and the known crosstalk among the multiple layers of biological regulation, the orchestrated analysis of the different omics levels remains essential. Thus, the study of deregulated pathways and the implementation of integrative systems biology approaches seems logical and sought after techniques (Auffray et al., 2010; Norris et al., 2017; Parker et al., 2019; Shafi et al., 2019; Myall et al., 2021). These approaches have been highlighted by their potential to better understand the complex, albeit inevitable, interactions among different omics data. Ultimately, systems analysis aims to elucidate the regulation of pathways that might underpin cause and effect factors and improve the understanding of systems behavior by providing more accurate models of a determined condition of interest.

Although integrative systems biology approaches have been applied to individual studies by performing a variety of high-throughput omics approaches and analyzing multiple layers of gene regulation data (genetic variants, RNA transcripts, DNA methylation profiles, protein concentrations, chromatin marks) (Boeing et al., 2016; Saha et al., 2018; Xicota et al., 2019; Mair et al., 2020), the possibility to sum-up and analyze publicly available data generated by different scientific groups from individual omics approaches through multi-study meta-analyses may not only increase the statistical power of the outcomes but enhance and complement the biological knowledge through the re-analysis and integration of large-scale data; thereby highlighting significant but previously undetectable molecular links.

**FIGURE 1:**
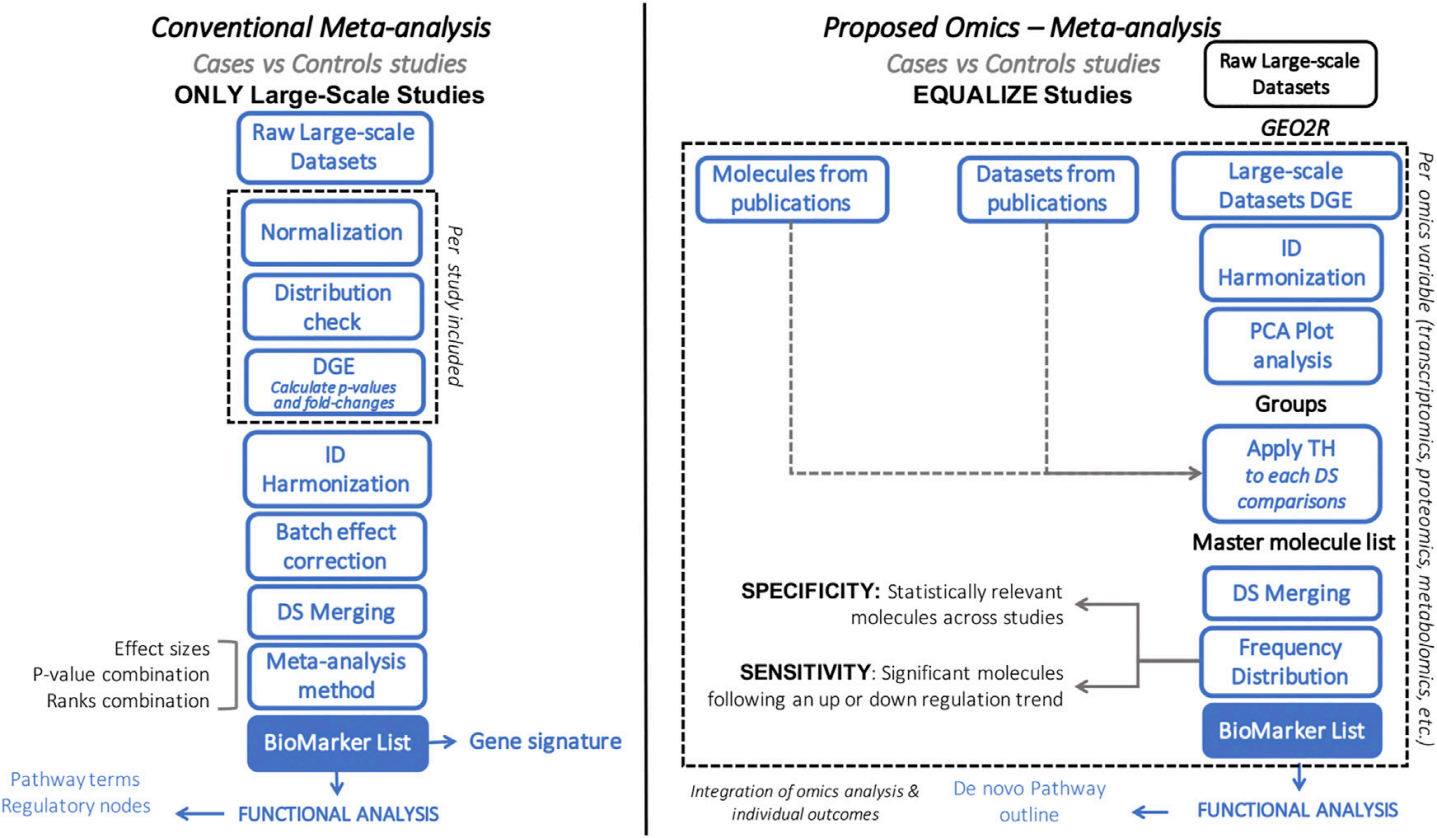
Flowchart comparing conventional (left) vs. our proposed (right) meta-analytic approach. Blue box represents similarities between approaches. Dashed line highlights the main differences between approaches.

Various methodologies are available to pursue systems biology analyses, each of which follows different strategies, with associated limitations and outcomes (Table 1) (Xia et al., 2013; Rohart et al., 2017; Argelaguet et al., 2018; Forero 2019; Singh et al., 2019; Winter et al., 2019; Zhou et al., 2019; Pang et al., 2020; Toro-Domínguez et al., 2020; Yang 2020; Zhou et al., 2020). Here we aim to describe the Harmonized Holistic (HH) meta method, a simplistic, flexible, adjustable, and scalable methodology (limited only by the availability of omics data) that can go from single omics to multi-omics analyses (Figure 1). Our methodology is not based on a computational approach, it is a meta-analysis based on case and control comparisons of pre-processed data per study. The basis of the data to perform integrative approaches is of importance, and there is where this meta-analysis approach gears towards allowing heterogeneous omics data integration. It can integrate unmatched mRNA, miRNA, DNA methylation profiles, protein, metabolites information from independent studies that meet the inclusion criteria of a specific research question. By considering the commonalities of the differentially expressed molecules across studies, the HHmeta method circumvents the variable depth of data produced by different measurement technologies (i.e., Microarray, RNAseq), as well as by high and low-throughput studies. This approach provides a ranking system that goes beyond the *p*-value and log2-Fold Change significance filtering, by defining the molecules with a significant and consistent trend in regulation among the different studies analyzed. Ultimately, this approach leads to the identification of pathways that can be fed and confirmed with the different omics data analyzed, which validates the outcomes and increase the significance of the identified targets (see Graphical Abstract in Supplementary Presentation S1).

**TABLE 1:** Comparison of available integrative systems biology methodologies.

Methodology	Strategy	Outcome	Limitations	References
HHmeta method	Meta-analysis of Differentially Expressed molecules from omics data. Data from different platforms (e.g. RNAseq, microarray) can be integrated. Biomarker list generation by ranking the frequency distribution and contextualization of molecules into pathways	- Integration of omics Biomarker lists and contextualization into pathway maps	- Depends on availability of the data	Cervantes-Gracia et al. (2021); current paper
		- Novel hypotheses from the Biomarker list	- Relies on previous knowledge	
		- Novel hypotheses from the Main deregulated pathways	- Gaps prevail across the pathway maps	
		- Better understanding of the disease/condition of interest	- Molecules without a defined function or interaction are not mapped	
Network meta-analysis	Meta-analysis of transcriptomics data by including Differentially Expressed comparison analysis per independent study	- Differentially Expressed Gene list based on meta-analysis of independent experimental studies - GSEA.	- Do not focuses on integrate different omics	Winter et al. (2019)
			- Focus on obtaining signatures/biomarkers	
MetaPCA	Meta-analysis of transcriptomic or epigenomic datasets through identification of a common eigen-space for dimension reduction	- Clusters and Patterns of gene expression profile	- Do not focuses on integrate different omics	Kim et al. (2018)
		- Robust to outliers	- Focus on obtaining signatures/molecular patterns	
MINT	Independent omics studies integration based on similar biological questions	- Identification of reproducible biomarker signatures	- It can only include studies with a sample size bigger than 3	Rohart et al. (2017)
	Allows supervised and unsupervised frameworks. It is a PLS-based method to model multi-group (studies) data		- Focus on obtaining signatures/biomarkers	
NetworkAnalyst	Gene expression profiling, meta-analysis and systems-level interpretation	- Creates and visualizes biological networks	- Format of gene expression profiles outside the application	Zhou et al. (2019)
		- Web-based meta-analysis of gene expression data	- Integration of transcriptomics studies	
		- Comparison of multi gene lists generated outside the tool		
		- Identification of shared and unique genes and processes, through multi-list heatmaps and enrichment networks		
Mergeomics	Multi-omics association data, pathway analysis and functional genomics, analysis. It corrects for dependencies between omics markers. Based on pathway or network-level meta-analysis	- Identification of key drivers of a disease and causal subnetworks for specific conditions	- Format of gene expression profiles outside the application	Arneson et al. (2016)
		- Single dataset: causal network or key regulatory genes can be identified	- Based on comparison files: Cases vs controls	
		- Multiple dataset (same or different data type): meta-analysis, causal networks, key regulatory genes		
		- Groups of disease associated genes: key regulators, condition sub-networks, gene sets association with other conditions or organisms		
INMEX	Meta-analysis of multiple gene-expression datasets that allows integration of transcriptomics and metabolomics datasets	- Data preparation	- Limited to integration of transcriptomics and metabolomics	Xia et al. (2013)
		- Statistical analysis: multiple datasets combination based on *p*-values, effect sizes, rank orders and other features		
		- Functional analysis and ID combination between genes and metabolites		
DIABLO	Multi-omics integrative, holistic and data-driven method	- Identification of known and novel multi-omics biomarkers	- Batch effect analyses in each dataset are needed prior to integration	Singh et al. (2019)
		- Identify correlated variables within omics datasets from the same samples	- Integration of different omics dataset from the same biological samples	
			- Focus on obtaining signatures/biomarkers	
MOFA	Unsupervised identification of principal sources of variation among multi-omics datasets	- Identification of factors specific to data modalities and common within multiple molecular layers	- Analysis and integration of different omics datasets from the same biological samples. Similar to DIABLO, JIVE, PARADIGM or MCIA.	Argelaguet et al. (2018); (Subramanian et al., 2020)
			- Linear model, thus, non-linear associations might be missed	
Ingenuity pathway analysis (IPA)	Multi-omics pathway analysis tool	- Building of networks to represent biological systems	- Commercial	Ingenuity Pathway Analysis tool (IPA; QIAGEN Inc., Germantown, MD, USA, https://www.qiagenbioinformatics.com/products
		- Pathway analysis and association of processes activation or inhibition in a specific condition	- Do not generate meta-analyses	
		- Identification of novel targets	- Un-reproducible results	
		- Comparison across multiple analyses. Similar to Pathway studio (Elsevier)	- Based on computational approaches	

We have previously explored our methodology in several iterations and proved its potential in a variety of disease settings (Fernandes and Husi 2016; Cervantes-Gracia and Husi 2018; Fernandes et al., 2018), however the ranking system was not properly established. Here, we 1) consolidate the final optimized pipeline and 2) apply this framework to 6 DLBCL (Diffuse Large B Cell Lymphoma) datasets (DS) from different studies and sources (tumoral tissue vs. b-cells). We aimed to identify the DS that indeed showed a potential correlation to further identify altered pathways coming only from B-cell deregulation (Cervantes-Gracia et al., 2021; Sheppard et al., 2018). The identified pathways represent the common deregulated processes found within the different DS included in the downstream analysis and delimit their significance by the outlined trend of the pathway identified. The outlined pathways can be further fed with different omics data by complementing with the variables that correlate with the outcomes from the different omics levels. This analysis shows the potential of the presented methodology to not only identify potential biomarkers but also deregulated processes with a notable trend providing data-driven hypotheses that have either already been validated or that better yet have not been associated with the disease and need further validation.

## 2 Materials and Methods

The cornerstone of this methodology is for each group of interest to be compared to their most appropriate controls. Hence, the integration of large-scale DS from a variety of publications relies on keeping the study groups per publication intact. Thus, the basis of the methodology is set on statistically significant molecules and their ratio-metric values (e.g., log-fold change) per comparison, identifiers curation through a unifier database, data-format and structure, outlier detection, as well as group re-stratification. This methodology has been previously performed manually (Cervantes-Gracia and Husi 2018). The aim of this work is to describe and summarize the whole procedure into a formula that statistically ranks and explains the significance of the molecules included in the biomarker list. Here, both the manual approach and the frequency score (FS) index are presented.

The particular molecules under study (e.g., miRs, mRNA, proteins) are individually processed and meta-analyzed before the multi-omics integration and analysis is performed. In this methodology, each molecular type has its own comparison matrix where the large-scale studies are merged. Thus, every individual DS comparison (case vs. control) has the main molecules included in the analysis facing each other. The output of the HHmeta method is divided on biomarker identification and functional analysis. The methodology outline is divided into four main sections: Data collection, Data correlation and structure, Grouping and Biomarker discovery and Data integration and Functional analysis.

### 2.1 Data Collection

High-throughput DS from publications can be collected from public repositories such as GEO NCBI, ExpressionAtlas and ArrayExpress from EMBL-EBI databases for raw and processed omics data and SRA for raw sequencing data. Specialized databases exist for the different omics data. PRIDE, Peptide Atlas, ProteomicsDB, GPMDB, JPOST repository, MassIVE, PAXDB for proteomics, MetaboLights, MetabolomeExpress, MetabolomicsWorkbench, GNPS for metabolomics and EGA, EVA for genomics are examples of available omics databases (Carroll et al., 2010; Deutsch 2010; Coutant et al., 2012; Wang et al., 2012; Vizcaíno et al., 2013; Fenyö and Beavis 2015; Lappalainen et al., 2015; Kale et al., 2016; Sud et al., 2016; Wang et al., 2016; Wang et al., 2018; Samaras et al., 2020; Watanabe et al., 2021). Platforms like Omics discovery index (OmicsDI) exist, where biological and technical metadata from public omics datasets are stored and standardized through an indexing system to enable access, discovery and broadcasting of omics datasets (Perez-Riverol et al., 2017). In terms of cancer databases TCGA, COSMIC, OCCPR and ICGC are distinguished high-throughput data repositories. Data can also be collected directly from the literature. The DS collected can be derived from entirely unmatched sources (e.g., DNA, RNA, protein), different platforms (e.g., Microarray, RNAseq), and samples (e.g., tissue, blood, urine).

Here, the example shows the analysis of DLBCL and the potential to correlate and find the common and significant molecules and deregulated mechanisms across expression profiles from tumoral vs healthy samples. The following DS (gene expression profiles) were retrieved from GEO (NCBI) database (Clough and Barrett 2016): GSE9327 (tumoral tissue vs healthy tissue; CNIO Human Oncochip), GSE32018 (tumoral tissue vs. healthy tissue; Agilent), GSE56315 (tumoral tissue vs. healthy B-cells; Affymetrix), GSE12195 (tumoral tissue vs. healthy B-cells; Affymetrix), GSE2350 (tumoral B-cells vs. healthy B-cells; Affymetrix), GSE12453 (tumoral B-cells vs. healthy B-cells; Affymetrix).

### 2.2 Data Correlation and Structure

This module comprises three steps: DS group comparison, Data ID harmonization, and Data Merging within and across DS comparisons.

#### 2.2.1 Datasets Group Comparison

This step represents the first statistical evaluation embedded within this methodology. Here we rely on pre-processed and normalized available DS; raw data can also be considered. Raw samples need to be normalized individually to be further statistically assessed and generate ratio-metric values. Differential expression analysis of the GEO DS collected are performed through GEO2R, a web-based tool that includes GEO Query and Limma Bioconductor packages and performs multiple-testing correction through Benjamini–Hochberg false discovery rate method as a default (Benjamini and Hochberg 1995; Gentleman et al., 2004; Smyth 2004; Sean and Meltzer 2007). Data collected directly from the literature already provides ratio-metric values to integrate into the correlation matrix.

#### 2.2.2 Data ID Harmonization

Given that different experimental platforms (e.g., different microarray technologies, RNAseq) and functional analysis tools usually produce and require unique identifiers, there is a need for standard names for each type of molecule (e.g., transcript, protein) under study. In order to be able to correlate the ratio-metric values of the molecules shared across every DS comparison included, and reduce data redundancy within studies, the DS needs to be mapped to a common identifier (e.g., Uniprot or PADB identifiers). Furthermore, a unifier that consolidates the different accession numbers and identifiers can be of great assistance. The PADB database was established by H. Husi (Husi 2004) as a unifier database for molecular data and it has been the reference database for several of our studies (available by request). PADB has been continuously curated and updated over the last 20 years. It contains old and recent identifiers from multiple databases and platforms that have been assigned to the molecules through time. This database clusters identifiers from a variety of databases and platforms (Ensemble, Genenames, RefSeq, Uniprot, Swissprot, Agilent, Affymetrix, Illumina, and others), and provides a unique unifier ID that maps the molecules to all these reliable identifiers, allowing further data merging and analysis through a variety of tools. Uniprot database and BioMart also offer the option to retrieve alternative identifiers (e.g., Ensemble, Genenames) for molecules of interest or by downloading the complete file to index by any cross-indexing tool (Smedley et al., 2009; Consortium et al., 2021).

To cross-index and further merging of accession numbers, the in-house software AWASH was used. It is a text manipulation software that performs data cleaning and merging by using either a single file or multiple files, the latter based on a parent file as a reference for further indexing on dependent files. To perform the indexing, a Master file (containing the identifiers from the common database chosen and the accession number of the DS in question) and a Child file (different files for each DS comparison containing all accession numbers, statistics, and ratio-metric values) are needed. The input files should be in Tab- Separated Values (TSV) format. After indexing, each Child file accession number will be associated with a common unifier ID and alternative identifiers.

#### 2.2.3 Data Merging Within and Across Datasets Comparisons

Often within large-scale DS, there is more than one probe-set and values for the same molecule. Thus, the significant (e.g., FDR/*p*-value) and ratio-metric (e.g., Fold change) values from molecules with the same unifier ID can be either merged or one can keep only the probe-sets that have the most significant values, as long as the same method is followed for each DS comparison merging. Every DS comparison should only contain one ID for each of the molecules within it. AWASH software can be used for this purpose based on the common unifier IDs and a Masterfile containing each of the DS comparisons.

Once all cleaned, all the DS comparisons from the same molecular type are merged into a matrix based on a list of unifiers reported among all the DS comparisons. The identifiers from all DS comparisons would follow the same order. Thus, the same molecule would be facing each other across the different DS comparisons, a fitting format for further analysis. In the manual approach we only focused on the molecules below a *p*-value of 0.05 for all the DS comparisons included independently of their ratio-metric value. However, when calculating the FS index score, since it takes care of the filtering, there is no need of applying cut-off at this step. In case of a statistically poor dataset, where adjusted *p*-values were greater than 0.05, the unadjusted values should be used.

### 2.3 Grouping and Biomarker Discovery

Dimensionality reduction facilitates analysis and visualization of high-throughput data. This methodology relies on principal component analysis (PCA) to cluster and interpret large-scale DS comparisons. This step represents the 2nd statistical evaluation within this approach. PCA plots allow the identification of outliers, but most importantly it provides a confirmation of the DS comparisons that group together and can be further integrated to perform further analyses. The latter will reduce bias and act as batch effects removal. In order to avoid gaps in the data-matrix and misleading clustering, this analysis should only include and compare the expression-level differences of the molecules analyzed and shared among all DS comparisons.

The 3rd statistical evaluation is founded on pattern matching and centroid clustering to obtain the biomarker list. In the manual approach, DS statistical pattern recognition is based on correlation analysis to generate the biomarker list. Here, unique thresholds (TH) are applied to each DS comparison (THs might vary across DS) depending on the number of their deregulated molecules (where more than 10% of deregulated molecules can give a hint of something off being compared within a DS). Only molecules with significant *p*-values and log 2 fold-change (FC) (above 1 or 2 depending on the DS comparison) are included. A master molecule list (MML) is created containing all the significant molecules reported within THs per DS comparison without repetitions. The MML is used to merge all DS as described above to perform cross-correlation analysis and obtain the biomarker list.

To calculate an accurate frequency distribution manually per molecule, the trend in regulation is determined, taking into account the total count per molecule (TPM), to avoid bias. The regulation trend is described as “Up” or “Down”; molecules reported equally “Up” or “Down” regulated (i.e., 50% up and 50% down regulated) among the DS comparisons are removed. TPM represents the number of times a molecule is analyzed across all the DS comparisons included in the data-matrix. The latter highlights the point that every platform might have different molecular depth, thus, if a molecule is not analyzed in one platform it doesn’t mean it is not significant. Consequently, a biomarker list is created and can already be validated. This list is the core of the next functional analysis.

Regarding the FS index, it was developed to generate the former biomarker list from all values across all DS comparisons, without the need of applying individual TH (see below). The FS index is simply based on the log_2_FC trend per molecule and whether these are significant or not. The formula is as follows:

$$FS=\frac{\left|\sum \left(up\right)-\sum \left(down\right)\right|}{\sum \left(up\right)+\sum \left(down\right)}\;\times \;\frac{\sum \left(significant\right)}{\sum \left(all\right)}$$

From all the DS comparisons, the absolute value of the sum of DS comparisons with up-regulated values (log_2_FC > 1) is subtracted from the number of DS comparisons with down-regulation (log_2_FC < -1) and divided by the sum of the number of times a molecule was up and down regulated (log_2_FC > 1 and <−1). This value is then multiplied by the value obtained from the sum of the number of DS comparisons that have significant values (adjusted *p*-value <0.05) divided by all the DS comparisons that have the specific molecule in question analyzed.

**FIGURE 2:**
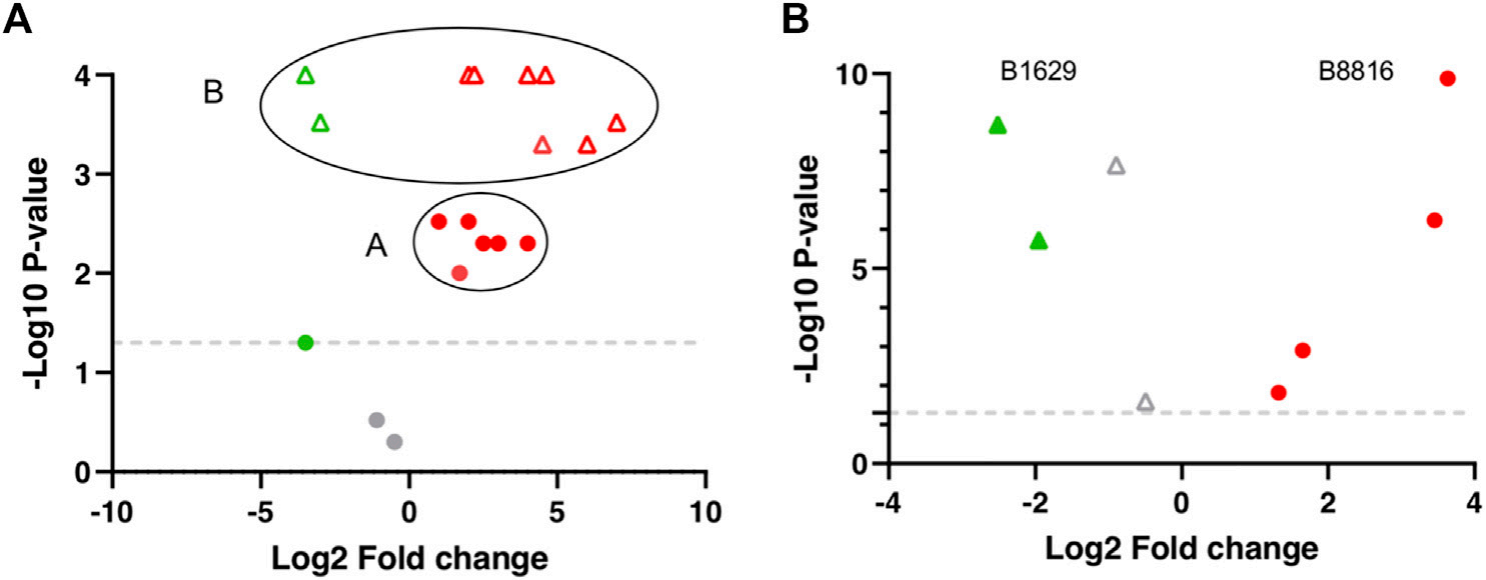
Theoretical and Real centroiding clustering example visualized in a Volcano plot. **(A)** A and B represent 2 different molecules. Red and gray circles represent molecule A distribution from the different dataset (DS) comparisons. The majority of molecule A values cluster regarding regulation, log2FC, and *p*-values (Red circles). Gray circles represent molecule A with non-significant *p*-values. Green and Red triangles represent molecule B distribution. All molecule B values are significant and cluster regarding regulation, log2FC, and *p*-values (Red triangles) but two DS comparisons (Green triangles). **(B)** B1629 and B8816 are real molecules within the DS matrix and represents an example of the distribution of 2 molecules from the biomarker list obtained through the FS index.

Additionally, the FS index includes an adjusted *p*-value and log2FC calculation. Here, the basis of the approach is centroiding clustering and it applies to all molecules individually. Molecules with *p*-values and FCs for all the included DS can have significant or non-significant values. To visualize the distribution of the data, molecule sets −log (10) of the *p*-values and the log (2) of the FCs can be plotted to get a Volcano plot (Figure 2). Here, centroiding clustering brings a logical solution to fuse the data, with the center being the optimal geometric location that minimizes the distance to all datapoints. In an ideal situation the graph (Figure 2A) would have a small group of datapoints close to each other for a given molecule (similar *p*-values and similar FCs). The center-value will then provide a new *p*-value and FC value. To obtain the adjusted *p*-value its easier than the adjusted log_2_FC value, since it does not have directionality; in this case an arithmetic mean is calculated from the −log10 *p*-values to obtain the adjusted *p*-value, since the non-transformed *p*-values are too small and here a TH does not apply, therefore significant and non-significant *p*-values are included and averaging these can introduce bias. Regarding the adjusted log2FC, a common mean calculation might give a biased result due to the possible large values with opposing trends that the different DS comparisons can have (Figure 2B). There are still several options one can follow, such as geometric weighted means, where a specific value is added to each log2FC, e.g., sample size; trimmed means where extreme values (outliers) are left out, and the mean is calculated from the values that remain (Lawson et al., 2012; Miao and Jiang 2014; Li X. et al., 2015). However, in this example and regarding the FS index calculation, only where >50% of the molecules follow the same trend (up or down regulation) with values above or below 0 molecule value were averaged, the rest of the molecules were excluded.

These data, plus the calculated FS index value, upgrade the score system and allow us to rank the molecules from the most to the least significant based on: total significance, trend (up/down-regulation), number of times a molecule was analysed and present an either up or down-regulation, adjusted *p*-value and adjusted log2FC, which sums up into the FS index score. The higher the score, the more significant a molecule is.

### 2.4 Data Integration and Functional Analysis

Despite the biomarker list potential to lead to new insights about the disease in question, the contextualization of these molecules can be even more informative. In this section of the approach, the integration of the biomarker list through enrichment analysis is performed. Functional analysis through Cytoscape plug-ins ClueGO/CluePedia performs semantic clustering by assigning gene ontologies and/or pathway terms (KEGG, Wikipathways, Reactome) to the biomarker list, integrates them into functional networks and ties-in the molecules associated with each of the terms on the networks generated (Shannon et al., 2003; Bindea et al., 2009; Bindea et al., 2013).

This analysis will highlight the main deregulated processes that will be the center for further analyses. The biomarker list contains molecules with different frequencies and FS index scores, and results from the merging of the different DS comparisons. Several TH are applied to the biomarker list to determine the main deregulated pathways within it. The THs go from the most to the least significant and frequent molecules within the biomarker list. The TH end cut-off goes to a level where the processes identified through the analysis of the most frequent molecules are not lost but enriched and interconnected by the molecules from the different THs applied.

In order to visualize and underlie the main processes previously identified, pathway mapping is performed. It helps unravel the regulation and involvement of the molecules by placing them within their described position in the pathway of interest. This allows the accurate identification of deregulated processes by showing specific trends through the molecular interplay, hence the identification of key players involved in the pathophysiology of a disease. Pathvisio is a pathway-map editor of described and pre-assembled pathway maps (KEGG, Wikipathways, Reactome), it is a fine tool for integrative analysis since it handles gene, protein, and metabolite data and allows cross-mapping and integration through Bridgedb (van Iersel et al., 2010; Kutmon et al., 2015). The pre-assembled pathway maps will function as the sketch to base on to get novel pathway maps reflecting their regulation in the setting of interest. The pathways to be filled-in, edited, and integrated are the ones identified by ClueGO/CluePedia analysis. First, the molecules from the biomarker list are mapped into their specific position within these pathways to help keep the focus. Afterwards, irrespective of their logFC, all the molecules from the first data merging with a significant *p*-value are mapped as well in order to fill the gaps within the pathway map of interest and be able to identify trends in regulation.

To enrich and complement the processes of interest identified through Pathvisio, interactome analyses are performed on the biomarker list molecules. GeneMANIA (Multiple Association Network Integration Algorithm) generates networks that resemble molecular interactions classified into gene-protein interaction, co-expression, and localization, shared protein domains, and pathways (Warde-Farley et al., 2010). It provides the connections of the biomarker list molecules and predicts molecules associated with the input.

In addition, disease analysis to explore the former outcomes and their accurate association with the specific disease of interest is performed. Through DisGeNET, reported gene-disease associations from the biomarker list are identified (Piñero et al., 2015; Piñero et al., 2020). This step functions as validation by detecting the genes that have already been reported in the disease under study. DisGeNET also provides genes with gene-disease-association (GDA) scores, and the ones with a higher score can be used to enrich the pathway model by identifying their associations with the biomarker list molecules and thus, provide an extra focus on the pathways where these molecules play a role.

De novo pathway contextualization is produced by the integration of all the different results previously obtained. Since transcriptomic studies populate the databases and literature, these are the backbone of the methodology. When analyzing different molecular types, each OMIC layer validate and reinforce the focus on the deregulated mechanisms identified through transcriptomics analysis. miRNAs (miRs) and metabolites do not align with gene/proteins but can also be integrated into the developed model. In this case, cross-mapping can be carried out through “mode of action” by using either their targeted genes as a substitute ID (miR) or how they are produced (metabolites) using the associated enzyme to tie them into other OMICS data. By using the common unifier, it allows their correlation and mapping into the *de novo* pathway model described. CluePedia and MetaboAnalyst web-tools can serve this purpose by enriching miRs and metabolites respectively (Pang et al., 2020). In this example, only transcriptomics data is included.

## 3 Results

### 3.1 DLBCL Dataets Comparisons Correlation and Grouping

A total of 6 DLBCL gene expression profiles from human samples were identified through GEO, correlated, and meta-analyzed through the HHmeta method. From 6 GEO DS we ended up with 10 comparisons: GSE9327–1. DLBCL vs Healthy tissue; GSE32018–2. DLBCL vs. Healthy tissue; GSE56315–3. Plasmablast DLBCL vs. Plasmablast Healthy B-cell, 4. Centroblast DLBCL vs. Centroblast Healthy B-cell, 5. Centrocyte DLBCL vs. Centrocyte Healthy B-cell, 6. DLBCL vs. Healthy B-cells; GSE12195–7. DLBCL vs. Healthy B-cells; GSE2350–8. DLBCL vs. Healthy B-cells; 9. DLBCL CD19 B-cells vs. Healthy B-cells; GSE12453–10. DLBCL B-cells vs. Healthy B-cells. All molecules from the DS comparisons were mapped and indexed to PADB unifier ID. Only molecules/probe-sets with the most significant *p*-values were kept among the repeats found within each DS comparison. For the manual approach, DS comparisons were filtered by *p*-value (<0.05), regardless of their logFC value, and merged. When following the HHmeta method, no filtering is needed at this stage.

**FIGURE 3:**
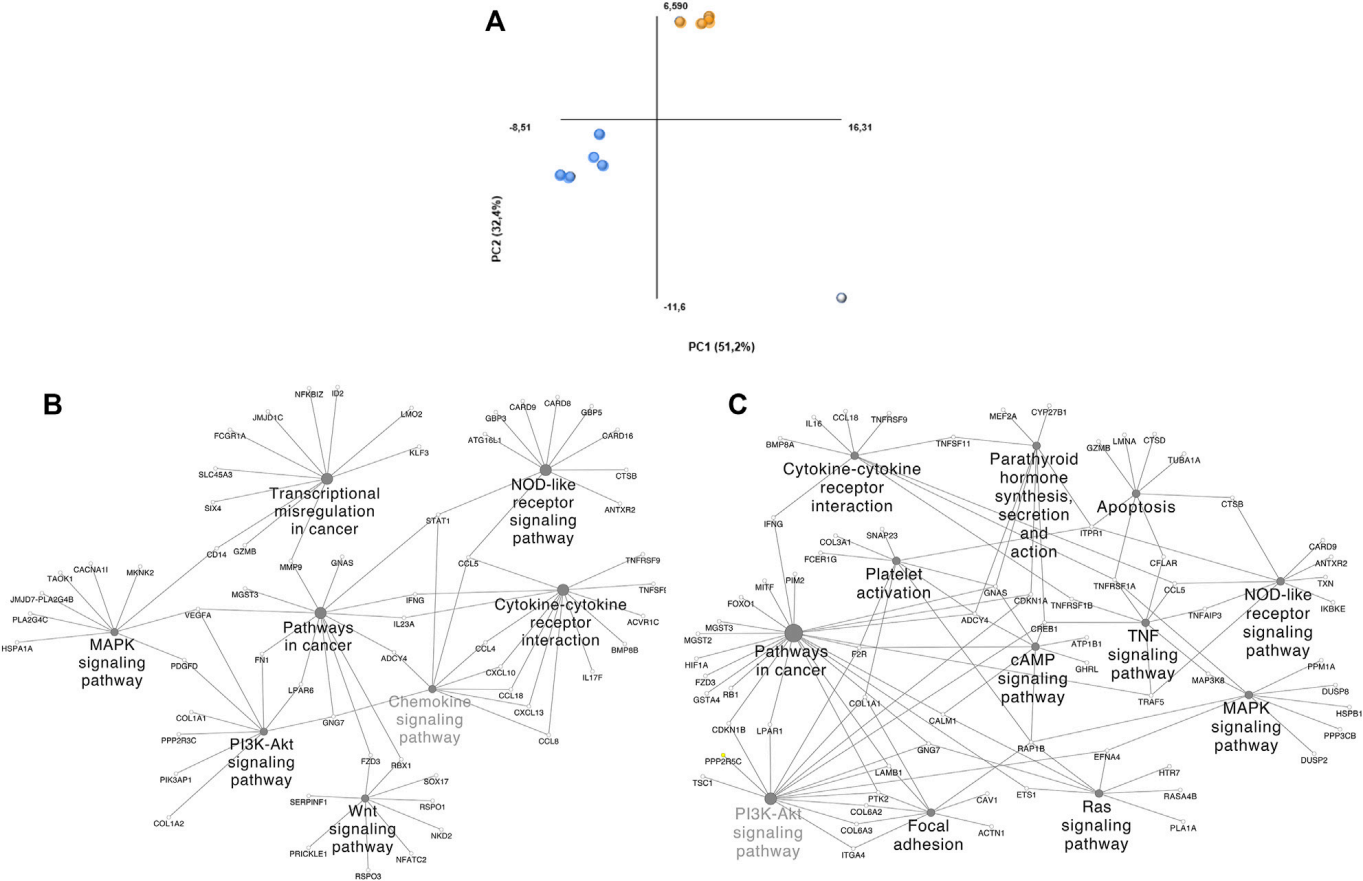
PCA plot grouping and ClueGO/CluePedia focus of our DLBCL cohorts. **(A)** Blue dots: Group 1; Orange dots: Group 2; White dots: Outliers. **(B)** ClueGO/CluePedia network created from the Manual approach biomarker list (66% threshold). **(C)** ClueGO/CluePedia network created from the FS index Biomarker list (0.75 Absolute FS index threshold).

Dimensionality reduction through PCA clustered 2 groups and identified a potential outlier (Figure 3A). DS 1, 2, 8, 9, and 10 (Group 1) and DS 3, 4, 5, and 6 (Group 2) were clustered together. Group 1 is composed of comparisons across healthy and tumoral tissue (DS 1 and 2), as well as healthy and tumoral B-cells (DS 9 and 10), however DS 8 compares both healthy B cells vs DLBCL tumoral tissue and also groups within this cluster. Group 2 contains 1 solely GEO DS (GSE56315) that is composed mainly by comparisons among specific tumoral tissue DLBCL subtypes and their matching healthy B-cell type, as well as the comparison of all of them together plus some unclassified DLBCLs and the complete population of healthy B-cells. Group 2 samples belong to patients under either CHOP or R-CHOP therapy. The highlighted outlier, DS 7 is composed of DLBCL tissue samples and healthy tonsillar germinal center, naive and memory B cells.

### 3.2 Biomarker List and ClueGO/CluePedia Functional Analysis

Once grouped, the following analysis focused only on group 1. For the manual procedure, *p*-value cut-off (<0.05) from the previous filtering was kept and log2FC cut-offs (>1 and <−1) were applied to each DS comparison. When applying the HHmeta method FS index formula (see above), no threshold is needed. Data merging allowed the comparison and consolidation of molecular regulation based on their frequency distribution. The resultant biomarker list from the manual procedure contains a total of 3,241 significant molecules, and from the FS index calculation, a total of 1,638 significant molecule (Supplementary Table S1). Table 2 represents the top up and down-regulated molecules from both biomarker lists group 1.

**TABLE 2:** Top deregulated molecules obtained with the Manual approach and FS Index calculation.

ID		Manual approach	HHmeta method		
CluSO ID	Gene name	Final Regulation	Adj. P.V. Mean	Log2FC Mean	FS Index Calculation
B2Q85	ITGA9	100	9.130E-23	4.29	1
B2O29	BIRC3	100	2.480E-05	2.19	1
BO058	HLA-DRB1	100	3.320E-03	2.04	1
BO135	BCL6	100	1.210E-04	2.01	1
B8773	LCE2D	−100	2.760E-03	−2.24	1
B9009	LPP	−100	4.687E-05	−2.25	1
BF875	SYCE1L	−100	1.270E-05	−2.29	1
B1137	ATP10D	−100	8.127E-08	−2.30	1
BL316	DNM1DN8-2	−100	3.360E-04	−2.40	1
BH305	TSPYL5	−100	4.326E-07	−2.49	1
B5415	FAM208B	−100	7.820E-07	−2.55	1
B7596	IGF2	−100	3.810E-08	−2.59	1
BO237	RET	−100	1.400E-17	−2.70	1
B8780	LCE5A	−100	1.660E-04	−2.87	1
B8612	KRTAP5-3	−100	2.240E-07	−3.00	1
B6621	GPR150	−100	3.360E-07	−3.01	1
B2W20	YES1	−100	2.380E-08	−3.45	1
B7559	IFITM5	−100	3.360E-07	−3.46	1

Through DisGENET one can already search for validation of genes associated with the condition in question within the biomarker list generated or target genes obtained from the functional analysis. In this example biomarker list genes with a FS index equal to 1 (439 genes) were input into DisGENET. As shown in Table 3, some have already been reported as related to DLBCL, either as e.g., biomarker, altered expression or genetic variants. DLBCL is a highly heterogeneous disease, thus it is important to bear in mind that in this example we didn’t segregate DLBCL by type (e.g., GCB, ABC), mainly because it was not specified in every dataset included. Therefore, the outcome of this analysis would mirror the core shared mechanisms among the DLBCLs analyzed in each of the different studies included in the meta-analysis of group 1. Also, within the genes found to be associated with DLBCL from the biomarker list, these might have been significantly deregulated in only one dataset because it was the only one analyzing this molecule, however this doesn’t rule out its importance since these can still interact with the main pathways further identified. For instance, genes involved in NF-kappa B pathway and TNF signaling, such as overexpression of BCL2 regulator of mitochondrial apoptotic pathway (Tsuyama et al., 2017), BCL6 proto-oncogene, essential for GC development and FBXO11 a tumor-suppressor gene that stabilizes BCL6, have already been related with DLBCL accelerated development and poor prognosis (Saito et al., 2007; Duan et al., 2012; Zhang et al., 2015). These, plus the rest of molecules covered by DisGENET provide validation of the molecular list we relied on for further functional analysis, and the meta-analysis itself.

**TABLE 3:** Top genes associated with DLBCL through DisGENET.

Gene	GDA Score	Association Type	Number of PMIDs
BCL2	0.4	Biomarker Altered Expression Genetic Variation	222
FBXO11	0.32	Biomarker Genetic Variation Causal Mutation	2
IRF8	0.32	Biomarker Altered Expression Causal Mutation	2
BCL6	0.1	Biomarker Altered Expression Genetic Variation	224
BIRC3	0.08	Biomarker Genetic Variation	8
HDAC9	0.07	Biomarker Altered Expression	7
ZC3H12D	0.05	Biomarker	5
LIG4	0.04	Biomarker Post-translational modification	4
HLA-DRB1	0.03	Biomarker Genetic Variation	3
PSIP1	0.02	Biomarker	2

Besides the already described molecules in the DLBCL setting, the biomarker list contains molecules that have not been related with the disease yet. As an example, one of the main deregulated genes with a higher frequency distribution score is TSPYL5, which was found with a downregulation trend in 4 out of the 5 DS comparisons included in group 1. TSPYL5 has been attributed a tumor-suppressive function, and its hypermethylation has been previously linked with several cancers (Kim et al., 2010; Fan et al., 2020; Huang et al., 2020). TSPYL5 suppression has been associated with PTEN overexpression and AKT pathway inhibition (Vachani et al., 2007; Jung et al., 2008; Kim et al., 2010; Fan et al., 2020). Interestingly, TSPYL5 inhibition has been attributed to overexpression of miR-483–5p and miR-629. In prostate cancer it’s been recently proven that miR-483–5p antagonization through the long non-coding RNA LINC00908 lead to an upregulation of TSPYL5, inhibiting prostate cancer progression (Fan et al., 2020). miR-629 overexpression has also shown the ability to promote proliferation, migration and invasion in ovarian cancer by directly inhibiting TSPYL5 (Shao et al., 2017). Although a specific role in DLBCL has not being described yet, these results open a potential novel regulation of carcinogenesis in this setting. ATP10D is also within the genes with a higher FS index score. Although its association with DLBCL hasn’t been described yet, its downregulation has been significantly correlated with poor non-small cell lung cancer survival (Fusco et al., 2018). It belongs to a subfamily of P-type ATPases that play a role in phospholipids translocation, and its being specially associated with sphingolipids and ceramid plasma levels (Hicks et al., 2009). Sphingosine-1-phosphate (S1P) sphingolipids are considered signaling molecules involved in activation of carcinogenesis pathways and have been previously linked to increase lung cancer risk (Furuya et al., 2011; Alberg et al., 2013). These are interesting hypothesis that haven’t been explored yet that by following our unbiased method could be highlighted. Examples like these can already be validated, adding to the main DLBCL pathway mechanisms.

### 3.3 ClueGO/CluePedia Functional Analysis

To distinguish the association amongst the biomarker list molecules and determine their shared pathways and processes, ClueGO/CluePedia analyses were performed (Figures 3B–C). The main processes showing interconnectivity between the molecules from the biomarker list of both, the manual approach and FS index score were MAPK, PI3K, TNF, Ras and B-cell signaling pathways, cytokine-cytokine receptor interaction and chemokine signaling pathways, among others. These pathways show high interconnectivity and potential involvement of MMP9, STAT1, TNFRSF1A, NFKBIA, EFNA4, CCL5, RAP1B. Networks in Figures 3B,C are similar, even though the HHmeta method does not follow thresholds and has an extra layer of significance raking through the FS index score calculation. However, since the most significant molecules were shared among the biomarkerlists generated through the manual approach and the HHmeta method, the main deregulated processes remain.

All the main pathways highlighted through this analysis are somehow related with pro-survival signaling, and have been previously associated with DLBCL pathology, as well as with the heavy involvement and crucial role of the tumor microenvironment. Pro-survival effects via PI3K-AKT signaling pathway, Ras signaling pathway (Eric Davis et al., 2001; Davis et al., 2010; Miao et al., 2019), partly B-cell receptor signaling pathway and cytokine induction have been long correlated with DLBCL. TNF signaling pathway is known to be indispensable for survival of transformed B-cells. TNF-signaling pathway regulates NF-kappa B pathway and MAPK signaling pathway, which was also determined as significant in DLBCL (Webster and Vucic 2020).

### 3.4 Pathvisio Pathway Editing and Complementation

The main pathways determined by ClueGo/CluePedia are now involved in the following iteration step, which entails pathway overrepresentation analysis and visualization in Pathvisio. Pathways identified through ClueGO were displayed within the significant pathways determined by Pathvisio. In order to determine consistencies, inconsistencies, interconnected events, and fill the gaps within the signaling pathways visualized, the complete list of molecules with a significant *p*-value is used as an input. In this case, a total of 5,146 molecules from the FS index were analyzed. For instance, the total amount of molecules obtained from the FS index might differ from the ones of the manual approach. This because for the adjusted log2FC calculation, the FS index only takes molecules determined as up or down regulated for averaging (see section 2.3), leaving out some molecules with mixed values. The manual approach is more bias, where the arithmetic mean is calculated from all molecules with a significant *p*-value, without prior filtering.

**FIGURE 4:**
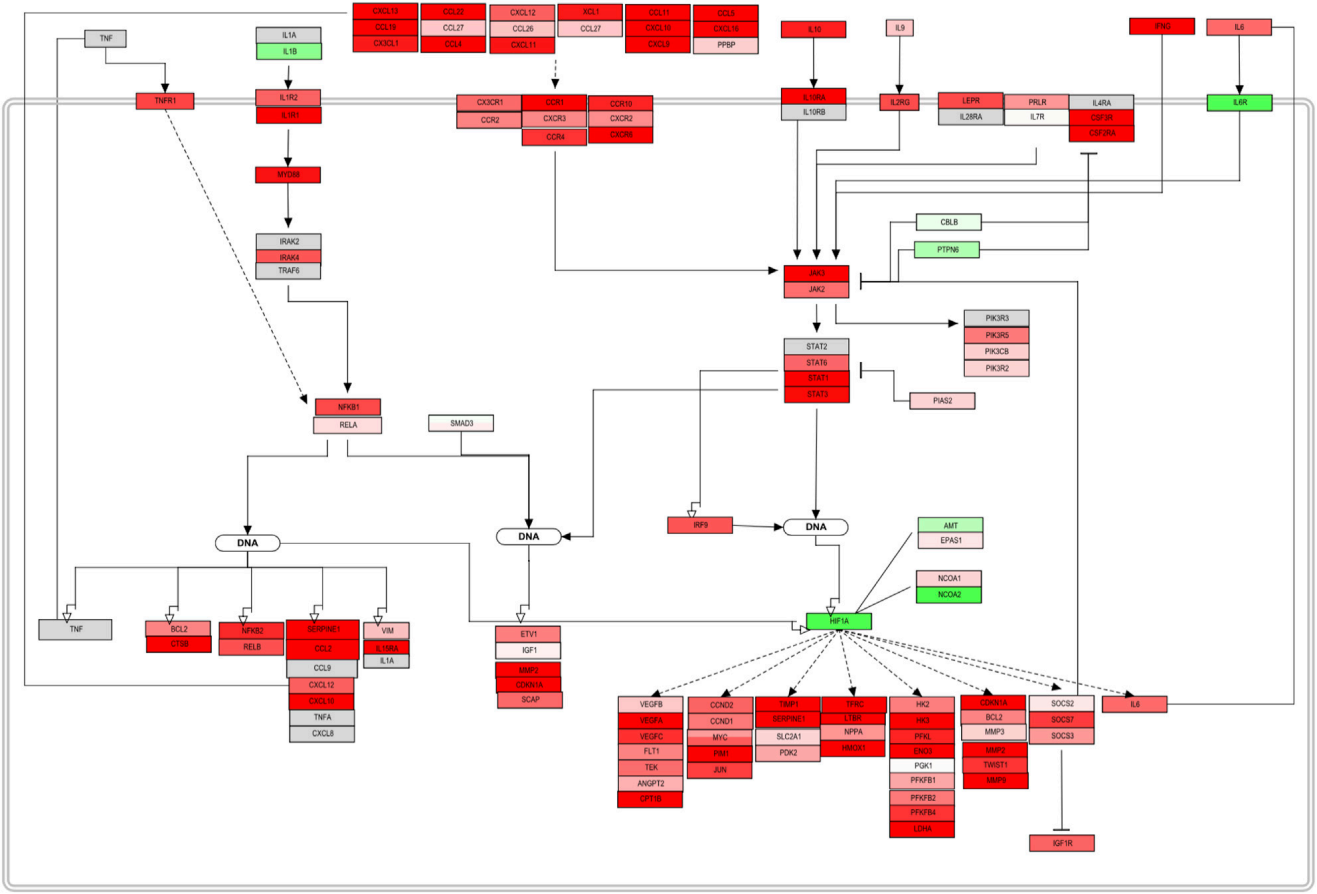
PathVisio edited pathway of the obtained biomarker list. NFKB and JAK/STAT section of the complete pathway map from Supplementary Figure S1. This section of a pathway contextualizes and represents the up-regulation trend of the molecules included in the map. Molecules with an adjusted *p*-value <0.05 from the FS index score calculation were included.

**FIGURE 5:**
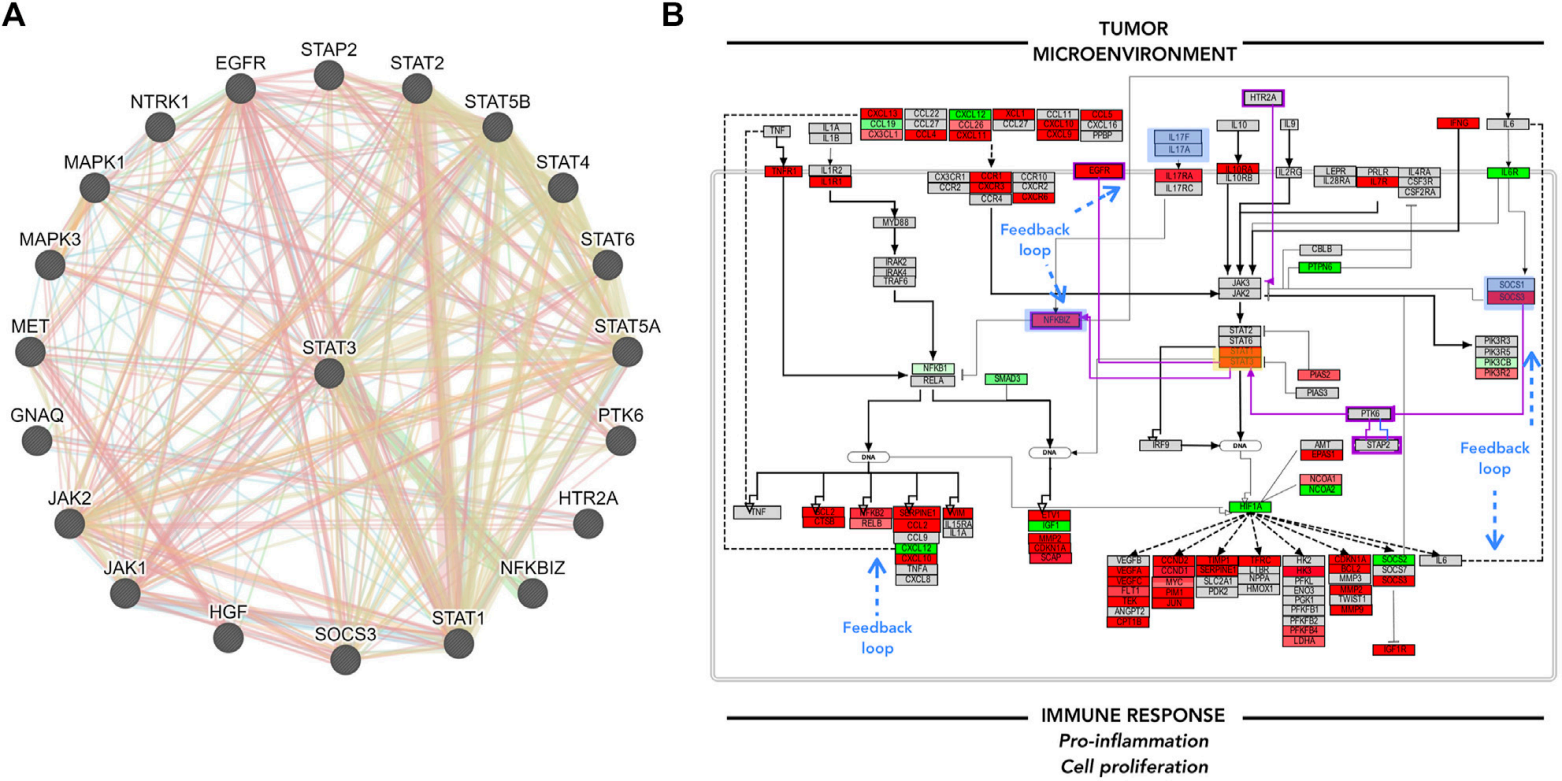
GeneMANIA focus and Pathvisio *de novo* pathway contextualization. **(A)** GeneMANIA results for STAT3; interactor molecules can either represent physical interaction (red), co-expression (purple), genetic interaction (green), shared protein domain (yellow). **(B)** Pathvisio NFKB and JAK/STAT signaling pathway section with added elements from GeneMANIA highlighted in purple; STAT3 analyzed gene highlighted in yellow box, blue boxes represent feedback loops. analyzed gene highlighted in yellow box, blue boxes represent feedback loops.

Once mapped, inconsistencies in the trend from sections or complete signaling events were removed from the original pathway maps. The complete pathway can be visualized in the Supplementary Figure S1. The original pathway maps from WikiPathways were redesigned to accurately contextualize the role and interplay of the molecules in DLBCL B-cells. Here, only a section of the assembled pathway is shown in Figure 4. From this signaling map, a prominent up-regulation of most of the elements involved can be seen. Chemokines, cytokines, and interleukin signaling demonstrate their involvement in the NF-KB and JAK-STAT pathway activation and therefore cell survival and proliferation. Several factors either display an opposite regulation or are absent within the biomarker list generated. However, the consistency along the pathway outline reflects an involvement of these processes in this B-cell malignancy. Feedback loops such as the one established by IL6, as well as regulators, such as PIAS, SOCS, and PTPN6 can already be spotted and represent potential hits to focus on further (Figure 5B).

GeneMANIA analysis can enrich the pathway maps from PathVisio. Several angles can be considered to add to the main hypothesis, for example, the top molecules from the biomarker list, the associated and clustered molecules obtained from ClueGO/CluePedia analysis, or the molecules of interest within the edited PathVisio pathway. Here, STAT3 was taken as an example to complement the pathway of interest, due to its downstream effects and the potential regulation of its activity. Figure 5. A. shows GeneMANIA results. Several molecules that were not included in the pathway map make an appearance. The connected molecules can either represent genomic interaction, shared protein domain, shared pathway, co-expression, co-localization, among other interactions. The additional interactors of STAT3 identified through GeneMANIA, such as NFKBIZ, EGFR, PTK6, and STAP2, complement the previous pathway, and the trend in consistency remains (Figure 5B). Here, one can hypothesize that the tumor microenvironment, such as T-cells, promote the chemokine/cytokine signaling in B-cells and lead to pro-inflammation, cellular proliferation and maintenance to some extent through JAK/STAT and NFKB signaling pathway constant activation. The interconnected and functionally correlated genes identified through ClueGO/CluePedia analysis, and some others integrated into the final edited pathway, have shown to be already associated with DLBCL.

## 4 Discussion

The decreased costs of high-throughput technologies have made the exploratory studies of complex biological traits, such as cancer, possible. Integrative omics approaches have been under the spotlight due to their potential to elucidate novel pathophysiological insights that better capture the complexity of molecular systems in a trait (Argelaguet et al., 2018; Kim et al., 2018; Zhou et al., 2019). Despite the increase in studies performing this type of analysis, efforts are still needed to better analyse and decipher the origin of complex diseases, for better diagnostics and discovering potential therapeutic targets, reviewed in (Yan et al., 2017; Karczewski and Snyder 2018). As a common characteristic, integrative methodologies rely on the identification of shared features across different large-scale datasets to further perform functional analysis. Nevertheless, one of the main elusive challenges that remains is the contextualization of the deregulated molecules; particularly in cancer where the high variability and intricacy of biomolecules involved can overwhelm meaningful readouts. In this setting, it is complex to identify commonalities among the systems altered by only looking at molecular signatures or protein-protein interactions, even within samples from the same cancer type. Thus, even though novel insights regarding potential correlations have been depicted across multi-omics studies (Zhang et al., 2013; Li J. et al., 2015; Mertins et al., 2016), the contextualization of how a molecule might influence or affect a system is still lacking. Our proposed methodology focuses not only on the identification of shared features, but also on their contextualization through pathway mapping. Approaches such as IPA, also focuses on the contextualization of features into pathway maps, however the lack of identifier curation, track and maintenance can result in poor reproducibility. In high-throughput studies the sample size is also an issue that might affect reproducibility and specificity. Usually, sample size increment correlates with higher reproducibility; it is equally responsible for an increase in false positives (Maleki et al., 2019). Thus, the fusion of integrative approaches, meta-analysis, associative data and enrichment methodologies gives an opportunity to boost the understanding, correlation and contextualization of potential molecules of interest affected in a disease. Moreover, one of the main hallmarks of our methodology is the enhancement of the statistical power of the biomarkers identified not only through the integration of high-throughput studies but also small-scale studies, which provides the focus on the pathway maps further described.

The majority of the available big data approaches rely on computational tools and therefore, the need of certain background to be able to perform these analyses. However, the HHmeta method provides a platform to not only perform an integrative meta-analysis, but also the opportunity for researchers lacking a solid background in bioinformatics to be able to perform an unbiased and straight-forward, but robust meta-analysis on pre-processed big data, to reach a logical and contextualized overview of the molecular interplay of a list of significant molecules related to an specific research question. In the example presented above, besides the identification of a deregulated and correlated set of molecules through out the analysis of different studies (Table 2), this methodology allowed their contextualization to identify potential processes and mechanisms involved in the disease (Figures 3, 4), and clarified targets influencing cell growth, survival and metastasis.

An interesting aspect that is commonly under-rated–but can influence downstream analysis and affect its replication–is the wide range of identifiers. Their constant update, reuse, un-usage, and the lack of unified efforts to both keep track and make mapping between different platforms available for the scientific community. Moreover, in order to merge datasets from different platforms and sources, the harmonization of identifiers is crucial. Thus, one of the solid basis and uniqueness of this methodology is its reliability on the PADB unifier database (see Methods section). Efforts have been previously made by others to address this issue (Gaj et al., 2007; Klimke et al., 2011), through BLASTx approaches (e.g., TargetIdentifier), linking annotations from different databases (e.g., DAVID) and trying to provide as much information as possible about IDs (Gaj et al., 2007), however data curation and constant update its still lacking. It has been noticed in pathway mapping that a great proportion of arrays become useless, mainly because there is no track of older IDs. Herein, PADB adds an extra quality-check to be able to rely fully on the available annotations and support the replication process. It enriches the downstream biological pathway map interpretation by retaining old identifiers for those molecules that currently have no annotation. PADB also allows cross-linking through species by its ortholog IDs (OMAP), enabling the identification of mechanisms that might be conserved across species through the downstream analysis.

Conventional meta-analyses apply several strategies to merge statistical measurements (i.e., *p*-value), and this is one of the main differences highlighted in the methodology presented here. Methods such as Fisher’s, Stouffer’s *Z*-test and Rank product are examples of popular statistical approaches to follow when performing meta-analyses to combine *p*-values of different studies, and their use depends on the meta-analysis goal (Zaykin et al., 2007; Hong and Breitling 2008). The former is based on testing the probability that different null-hypotheses, when combined, are statistically significant (Fisher 1992). However, here the proposed methodology relies on already statistically significant data for the manual approach; the FS index calculation (see section 2.3) relies on the number of times a molecule is significant, and the adjusted significance of a molecule is only one layer of ranking to consider. Therefore, in this setting Fisher’s method would not be the one of choice. In this methodology, the more a molecule is significant across studies the more likely it is that it is significant overall, regardless of the actual *p*-value, same with log2FCs, threshold values are used to set those boundaries. The significance of the molecules identified through this method is then corroborated by pathway mapping and other further analyses. Nevertheless, the HHmeta method and Fisher’s are similar in the principle of getting a new *p*-value (in our case also log2FC, plus the FS index) through the fusion of all the studies included. The main difference among conventional meta-analyses and our proposed methodology is that, by keeping the studies intact regarding cases and controls, and correlating the DS comparisons *p*-values and log2FCs, this methodology adds a layer of confidence regarding the comparisons made, allowing a primary correlation and clustering of studies through PCA plots.

PCA plot analysis have been used for the purpose of modelling the relationship between samples, to detect group differences and identify outliers and batch effects within a single high-throughput study (Ringnér 2008; Conesa et al., 2016; Merino et al., 2016; Todorov et al., 2018). Furthermore, there have been other integrative methodologies that have generalized PCA rationale to identify commonalities across different omics studies (Kim et al., 2017; Argelaguet et al., 2018). In contrast, here we apply PCA plot analysis to individual omics data types. Through this analysis, we were able to identify different groups and outliers from the initial high-throughput studies included in the analysis. This quality-check gives the opportunity to identify commonalities even amongst different samples, such as complete tumoral tissue and B-cells, by only including the molecules analysed in all the studies. It allows us to subtract commonalities across diverse studies, provided that the research question is well established. Even though the meta-analysis performed through this methodology is based on similar data, and therefore group of studies, it allows the comparison and identification of the relation between groups of different conditions (e.g., DLBCL vs. Healthy and DLBCL treated vs. DLBCL non-treated) opening new opportunities to identify specific responses and common enhanced pathways deregulated by the disease itself. Thus, to perform standard meta-analysis will be inadequate.

The major liability of the HHmeta method is that it is based on publicly available data. Thus, it is possible that the specific research question one wants to address hasn’t been covered by many research groups. The less high-throughput data sets available for a certain topic of interest, the less statistical power the data analysis would have. Moreover, if the available data is heterogenous, for example due to differences in the biology of the samples (treatments, stages of a disease, subtypes or sample sources), it makes the correlation even more complex, and the main question to address would need to change into a more general one, where commonalities can be depicted. Another weakness of the whole procedure is that the contextualization of the biomarker list relies on pathways previously described, so there will be gaps, molecules that do not map and unsolved questions. Despite these flaws, this method takes advantage of previous knowledge and uses it in the context of the specific topic of interest.

The substantial amount of data generated throughout the years represent a tool that can be somehow overlooked by the scientific community. For instance, good scientific practice can be enhanced by the screening, review and statistical analysis of previous studies performed in the field of interest to identify the gaps, commonalities and generating a better understanding regarding the behaviour of a system of interest, by feeding a potential model with what has already been proven and enhancing the generation of novel hypothesis to address by the inclusion of high-throughput data. The essence of the proposed methodology is the merging of independent statistical tests in an unbiased way, into a single test. It embraces the availability and basis of statistical analyses used in the big data field and utilizes their outcome to add to the statistical power of the data, resulting in a novel analysis approach. What sets the HHmeta method apart from the already available approaches are the basis of the data considered for merging, thresholding and its subsequent fusion and scoring system. Here, the manual and FS index approach are presented to highlight the main differences of what has been done before with the same ground basis as the FS index approach (FS index) which relies on a formula and adjusted values to produce similar results.

## 5 Conclusion

Studies in basic science are commonly hypothesis-driven and usually small-sampled. Likewise, and despite their exploratory nature, high-throughput studies tend to be biased to the resulting top deregulated genes. Therefore, novel findings require further validation, and here is where meta-analysis comes in handy. Even though the experimental design of different studies in essence is unique, meta-analysis methodologies have provided the opportunity to integrate the results of diverse and multiple studies addressing the same question, to enhance the statistical power of the results and therefore, the chances of finding true positives. In contrast to the meta-analysis methodologies already implemented in the big data-field, the methodology presented in this manuscript provides a simple, novel, unbiased, integrative and logical approach to not only meta-analyze single omics studies, but to integrate small and big data sets, as well as different omics studies. It includes quality checks to avoid batch effects, relies on a powerful cross-indexing unifier database and goes a step further by including associative data to aid for the identification and understanding of novel pathways and molecules involved in a specific disease. All in all, the current methodology provides novel hypotheses to further validate and a broader view of the system of interest, enhancing the outcomes generated through conventional meta-analysis.

## Data Availability

The original contributions presented in the study are included in the article/Supplementary Material, further inquiries can be directed to the corresponding authors.
