# Supplementary material for: Integrative OMICS Data-Driven Procedure Using a Derivatized Meta-Analysis Approach
Source: Front Genet. 2022 Feb 4;13:828786. doi: 10.3389/fgene.2022.828786 (PMC8855827; doi:10.3389/fgene.2022.828786)
Supplement: Supplementary file 2 [file Presentation1.PPTX]

## Slide 1
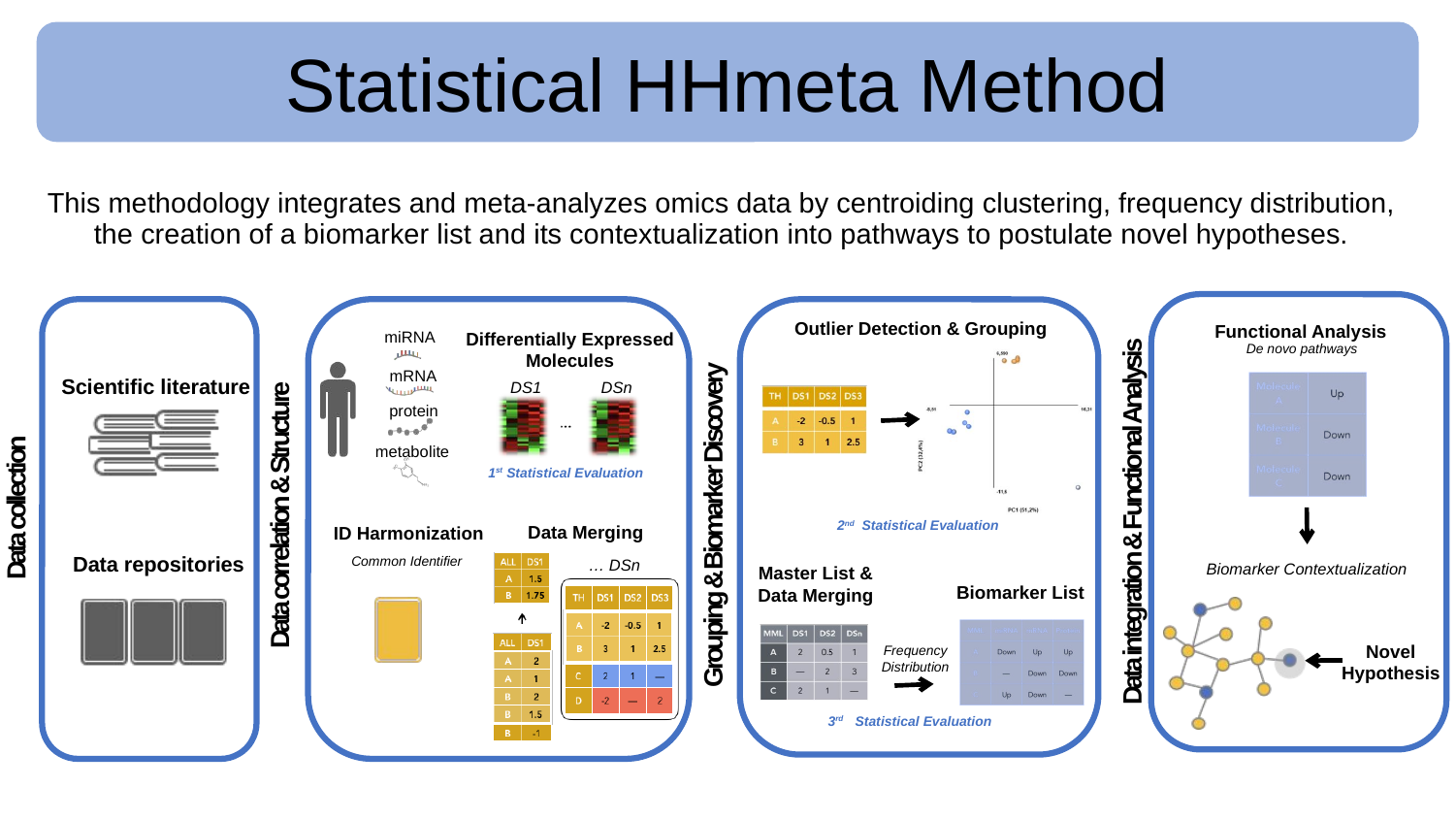

Statistical HHmeta Method
This methodology integrates and meta-analyzes omics data by centroiding clustering, frequency distribution, the creation of a biomarker list and its contextualization into pathways to postulate novel hypotheses.
Outlier Detection & Grouping
Functional Analysis
Data integration & Functional Analysis
Grouping & Biomarker Discovery
Differentially Expressed Molecules
miRNA
De novo pathways
mRNA
Scientific literature
DS1
DSn
Data correlation & Structure
protein
metabolite
Data collection
1st Statistical Evaluation
2nd Statistical Evaluation
Data Merging
ID Harmonization
Data repositories
Common Identifier
… DSn
Biomarker Contextualization
Master List & Data Merging
Biomarker List
Novel Hypothesis
Frequency Distribution
3rd Statistical Evaluation
